# Temperature-insensitive and low-loss single-mode silicon waveguide crossing covering all optical communication bands enabled by curved anisotropic metamaterial

**DOI:** 10.1515/nanoph-2023-0524

**Published:** 2023-10-06

**Authors:** Jinsong Zhang, Luhua Xu, Deng Mao, Yannick D’Mello, Zixian Wei, Weijia Li, David V. Plant

**Affiliations:** Department of Electrical and Computer Engineering, McGill University, Montreal, H3A 0E9, Canada

**Keywords:** silicon integrated photonics, anisotropic metamaterial, subwavelength grating, waveguide crossing, multi-band photonics device

## Abstract

We propose two designs of low-loss and temperature-insensitive single-mode waveguide crossing on silicon-on-insulator (SOI) platform with 415-nm operation bandwidth covering all optical communication bands. Both designs are enabled by subwavelength grating (SWG) modeled as an anisotropic metamaterial. The initial design applies straight SWG as the lateral cladding of the waveguide crossing to minimize the refractive index contrast and reduce the insertion loss (IL), but needs a relatively long taper. An improved design is then proposed where the curved SWG is introduced to replace the straight SWG to decrease the taper length and improve the performance. The waveguide crossing with the improved design achieves a calculated maximum IL of 0.229 dB and maximum crosstalk of −35.6 dB over a 415-nm wavelength range from 1260 nm to 1675 nm. The proposed devices are fabricated and characterized. Measured results of the improved design show a maximum IL of 0.264 dB and maximum crosstalk of −30.9 dB over a 230-nm wavelength range including O-, C-, and L-bands, which accord well with the simulation. Low temperature sensitivity has also been demonstrated in both simulations and experiments.

## 1 Introduction

The past decade has seen rapid advances in silicon-on-insulator (SOI) platform as a promising solution to large-scale integration of photonics devices. Considering the single-layer characteristic of the standard SOI platform, waveguide crossings are inevitable for on-chip routing. With the ever-increasing density and complexity of the photonic integrated circuits (PIC), low-loss silicon waveguide crossings are playing an increasingly important role in circuits design, e.g. large-scale silicon photonics switches [1, 2] and routing systems [3, 4].

Multiple structures have been proposed to achieve low-loss waveguide crossings on SOI. Vertical-coupling-based structures reduce the loss of crossings [5–7], however at the expense of increased fabrication complexity. Multi-mode interference (MMI) couplers have been utilized to decrease the loss of crossings while maintaining simplicity in fabrication [8–10]. With advanced design algorithms including particle swarm optimization (PSO) [11, 12] or Levenberg–Marquardt update [13], the loss per crossing can be reduced to <0.1 dB within C-band (1530–1565 nm). Moreover, it has been shown that a 1-D Gaussian beam can be synthesized by carefully manipulating the two or three lowest even-order modes to further minimize the insertion loss of a waveguide crossing [14, 15]. Such delicate designs achieve insertion losses <0.01 dB at optimum wavelengths.

Up to now, however, there is no previous study that has investigated an ultra-broadband low-loss single-mode waveguide crossing that covers multiple optical communication bands. For the past years, researchers have demonstrated such broadband designs of various SOI-integrated photonics devices including polarization beam splitters (PBS) [16], polarizers [17–20], polarization rotators [21], and 3-dB couplers [22]. These designs cover multiple or all optical communication bands (O-, E-, S-, C-, L-, and U-bands). Although broadband operation has been shown with multi-mode waveguide crossing [23], it is not practical for single-mode applications since a long taper is needed to convert single-mode waveguide to multi-mode waveguide.

Accordingly, this work aims to fill the gap in this area by proposing a broadband low-loss waveguide crossing on the SOI platform that covers all optical communication bands. Such a design would facilitate the development of multi-band PIC by expanding the broadband SOI device library.

As one of the commonly adapted structures for waveguide crossing, the MMI coupler makes use of the self-image effect to confine the modal electric field at the intersection. Despite the fact that MMI couplers are relatively broadband, it is shown in [8] that within a 60 nm band (1520 nm–1580 nm) the measured insertion loss (IL) per crossing varies by ∼0.2 dB. Similarly in a polarization insensitive crossing design based on MMI [10], the highest IL over a 90 nm band (1520 nm–1610 nm) is ∼0.2 dB more than that of the center wavelength 1550 nm for the transverse-electric (TE) mode. Even with the state-of-art optimization algorithms such as the Levenberg-Marquardt updates [13], one can observe that the IL declines to 0.5 dB with ∼230 nm bandwidth in simulation. Note that it is possible for such approaches that the performance over a large bandwidth can be improved by modifying the definition of figure of merit (FOM), yet it has not been demonstrated.

Therefore, we adopt a different idea to achieve the ultra-broadband operation. It is known that the large loss of a direct intersection of single-mode SOI waveguide stems from the high index contrast between the silicon core and the oxide cladding. The high contrast causes wide-angle spatial components in the mode that radiate away when the light enters the intersection area. In [24], a shallow-etched lateral cladding is introduced to decrease the index contrast, decreasing the IL to 0.16 dB. However, shallow-etch adds cost and complexity to fabrication. Hence, this paper proposes a single-layer design using an anisotropic metamaterial as the lateral cladding with simplified fabrication. The adopted metamaterial is the subwavelength gratings (SWG) [25–27]. The SWG consists of periodic structures with periods much smaller than the wavelength of the incident light. Since SWG exhibits capabilities in refractive index engineering and dispersion management, it adds degrees of freedom to the design and benefits various types of integrated photonic devices. The use of SWG has been reported in various fields, such as integrated devices in optical communication bands [27–29] and different types of chip-scale sensing [30–33]. Specifically for the topic of this work, SWG has been demonstrated in SOI waveguide crossings [23, 34], [35], [36] to maximize the transmittance. Besides index engineering, the anisotropic feature of the SWG has also been utilized to achieve ultra-broadband polarization handling on SOI [16, 17]. Our work is also inspired by the anisotropic modeling of the metamaterial. Similarly in [37–39], graphene-embedded silicon waveguides are adopted to achieve broadband TE-pass polarizers and polarization splitter and rotators (PSR) where the key factor is the graphene treated as an anisotropic metamaterial.

Unlike MMI-coupler-based crossings, such a design avoids the excitation of higher-order modes and then benefits the performance over a large range of wavelengths. Two designs are demonstrated, namely the initial design and the improved design. The improved design with curved SWG achieves a calculated maximum IL of 0.229 dB and maximum crosstalk of −35.6 dB from 1260 nm to 1675 nm, which covers all the optical communication bands. The device is also experimentally characterized that a maximum IL of 0.264 dB and maximum crosstalk of −30.9 dB is achieved within a 230-nm wavelength range that includes O-, C-, and L-bands. Meanwhile, such designs do not involve multi-mode interference or coupling, which makes the device insensitive to temperature changes. A low temperature sensitivity of our proposed waveguide crossing is shown in both simulations and experiments.

## 2 Design and simulation

### 2.1 Initial design

**Figure 1:**
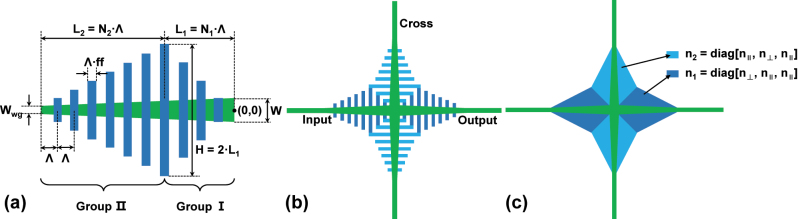
The schematics of the initial design. (a) Parameter definitions of a single arm. (b) The overall structure. (c) The illustration of how the SWG is regarded as anisotropic homogeneous mediums.

The initial schematic is shown in Figure 1. Since the proposed structure has 4-fold symmetry, we first define one of the four arms as in Figure 1(a), and then it can be expanded to its complete form in Figure 1(b). The basic component of the arm is a linearly tapered strip waveguide with a length of *L*, where the width changes from the *W*_wg_, the width of the connecting single-mode waveguide, to *W* at the intersection point (0,0). On top of it, two groups of SWG with the same pitch Λ and fill factor *f* are placed as the lateral cladding. Starting from the left-hand end, group II consists of *N*_2_ periods of SWG, with linearly increasing height from 0 to *H*, while the following group I contains *N*_1_ periods with decreasing height from *H* to 0. It consequently implies that *L* = *L*_1_ + *L*_2_ = (*N*_1_ + *N*_2_)*Λ. If the SWG is regarded as an anisotropic homogeneous medium as demonstrated in Figure 1(c), there are in fact two types of metamaterials due to the two different orientations of SWG [26, 40].

The diagonal relative permittivity tensors are given by [16]:

(1) $$\varepsilon_{1}=\mathrm{diag}\left[\varepsilon_{1}^{\mathrm{xx}},\varepsilon_{1}^{\mathrm{yy}},\varepsilon_{1}^{\mathrm{zz}}\right]=\mathrm{diag}\left[n_{⊥}^{2},n_{\|}^{2},n_{\|}^{2}\right],$$

(2) $$\varepsilon_{2}=\mathrm{diag}\left[\varepsilon_{2}^{\mathrm{xx}},\varepsilon_{2}^{\mathrm{yy}},\varepsilon_{2}^{\mathrm{zz}}\right]=\mathrm{diag}\left[n_{\|}^{2},n_{⊥}^{2},n_{\|}^{2}\right],$$

where the indexes of parallel and perpendicular polarization can be expressed (with zero-order approximation) as follows [26, 41]:

(3) $$n_{\|}^{2}=ff\cdot n_{\text{Si}}^{2}+\left(1-ff\right)\cdot n_{SiO_{2}}^{2},$$

(4) $$\frac{1}{n_{⊥}^{2}}=\frac{ff}{n_{\text{Si}}^{2}}+\frac{1-ff}{n_{SiO_{2}}^{2}},$$

We create 4 copies of the single arm via rotating it around the intersection point by 0°, 90°, 180°, and 270°, respectively. The combination of all 4 copies then forms the proposed structure. In order to properly connect the copies, the maximum SWG height *H* is set to 2 ⋅ *L*_1_. One detail to notice here is that the heights of SWG in group II need to be slightly extended by Λ ⋅ *ff* to avoid unmanufacturable small feature sizes. Since $n_{SiO_{2}}<n_{\|}<n_{\text{Si}}$ , $n_{SiO_{2}}<n_{⊥}<n_{\text{Si}}$ , a low-index-contrast waveguide profile is then created near the intersection area. In [24], it is stated that a low refractive index contrast reduces the diffraction loss and the back reflection in the non-confined area, hence the crossing region. Therefore with proper optimization of the SWG, the index can be finely engineered to maximize the transmission and minimize the crosstalk.

Prior to optimizing the geometries of the device, several parameters can be preset. Λ is set to 0.180 µm to ensure that the gratings work within the subwavelength regime at every target wavelength, of which the range is [1260 nm, 1675 nm]. We set *W*_wg_ = 350 nm so that the single-mode condition of the connecting waveguide is fulfilled for all the bands. In order to search for a global best FOM, we initially utilize PSO in 3-D finite-difference time-domain (FDTD) simulations with large mesh sizes to efficiently find suboptimal parameters, and then we carry out sweeps on key device geometries with finer meshes. We specifically use the Ansys Lumerical FDTD solver along with the built-in PSO algorithm to conduct the simulations. Note that the proposed devices work at TE mode, and are optimized with TE-mode simulations.

Commonly, the IL, the crosstalk, and the reflection are all important targets to optimize in the design process of a crossing. They are defined as:

(5) $$\text{IL}=-10*\log_{10}\left(P_{\text{out}}/P_{\text{in}}\right),$$

(6) $$\text{Crosstalk}=10*\log_{10}\left(P_{\text{cross}}/P_{\text{in}}\right),$$

(7) $$\text{Reflection}=10*\log_{10}\left({\hat{P}}_{\text{in}}/P_{\text{in}}\right),$$

where *P*_in_ is the input power, *P*_out_ is the power from the output port, *P*_cross_ is the power from the cross port, and ${\hat{P}}_{\text{in}}$ is the reflected power from the input port.

In our optimization process, the FOM is defined as the maximum IL over the whole wavelength range, i.e. we temporarily ignore the crosstalk and the reflection in the optimization. The parameter space is shown in Table 1. Note that for *N*_1_ and *N*_2_, since the PSO runs in continuous variable space, we set the final value as *ceil*(*N*_1_) and *ceil*(*N*_2_). The PSO runs with 30 generations and a generation size of 10.

**Table 1:** Ranges of parameters in PSO for the initial design.

Parameters	*N*_1_	*N*_2_	*ff*	*W* (µm)
Lower bounds	5	20	1/3	0.400
Upper bounds	15	39	2/3	1.00

**Figure 2:**
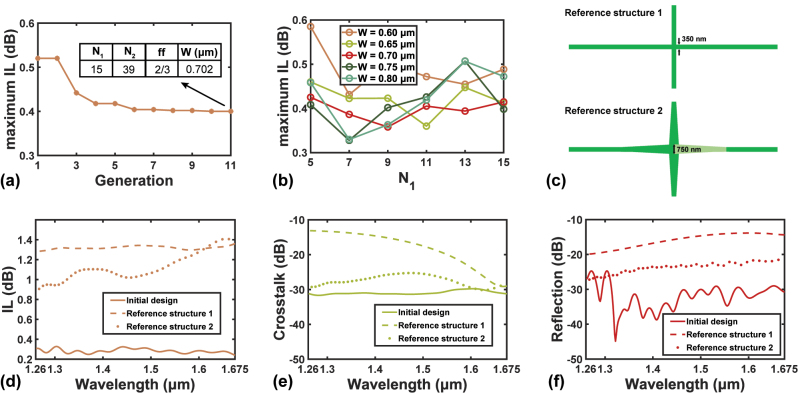
The simulation results of the initial design. (a) FOM trend of the PSO with the initial design. (b) Dependence of maximum IL on *W* and *N*_1_. (c) Schematics of two reference waveguide crossing structures (d–f) Calculated spectra of (d) IL, (e) crosstalk, and (f) reflection of the device with optimized geometry, along with the results of both reference structures in (c).

The simulation results are shown in Figure 2. Having conducted PSO, the FOM converges to 0.400 dB after 11 generations according to Figure 2(a), where the parameters at the last generation are also shown. In such a multivariate optimization task with little *a priori* information, the PSO proves to be an efficient tool to provide the designer with a decent starting point for further optimization.

For the next step of optimization, we perform parametric sweeps rather than PSO, and the number of mesh points per wavelength increases from ∼10 to ∼14 for more accuracy. We firstly fix *N*_2_ = 39 and *ff* = 2/3. The reason for fixing *N*_2_ is that the SWG of group II acts as a taper structure from strip waveguide to waveguide with SWG cladding. Therefore when *N*_2_ is large enough, it mainly determines the footprint of the device rather than the performance. Meanwhile, the fill factor *ff* reaches the upper bound 2/3 after convergence, and it is not feasible to further increase the value due to the 60 nm minimum feature size of the fabrication process. Therefore *ff* is also fixed as the upper bound to simplify the following optimization.

Then the device under various combinations of *N*_1_ and *W* are evaluated by simulation. The results are displayed in Figure 2(b). It is inferred that the PSO only reaches a local optimum, since the best FOM is achieved at *N*_1_ = 7 and *W* = 0.750 µm. The detailed device performances, including the IL, the crosstalk, and the reflection, are shown in Figure 2(d)–(f) as functions of wavelength. The IL over the 415 nm wavelength ranges from 0.237 dB to 0.328 dB, indicating a broadband operation. As for the crosstalk and the reflection, although they are not considered during the optimization, crosstalk <−29 dB and reflection <−24 dB are achieved. To better prove the functionality of SWG over the whole bandwidth, we also simulate two reference structures as shown in Figure 2(c). Reference structure 1 is a direct waveguide crossing of 350-nm wide waveguide, while reference 2 is a crossing with linear tapers from 350-nm to 750-nm widths. Reference 2 can also be formed by removing the SWG cladding in the initial design. Their simulation results are displayed in Figure 2(d)–(f) as benchmarks. The calculated results prove the validity of the proposed design with lateral SWG cladding, as the device maintains decent performance over all the optical communication bands, compared to direct crossings without the SWG.

### 2.2 Improved design

Although the initial structure has shown broadband features, there are several weaknesses about it. First of all, a long taper (group II SWG) is needed to achieve better performance, which increases the device footprint. Besides, the mode profile tends to change quickly as the light propagates near the intersection, because a 45° interface is inevitable between the two mediums as shown in Figure 1(c). Compared to the angle of common linear tapers on SOI, 45° is considered large and is likely to increase the loss of the device.

**Figure 3:**
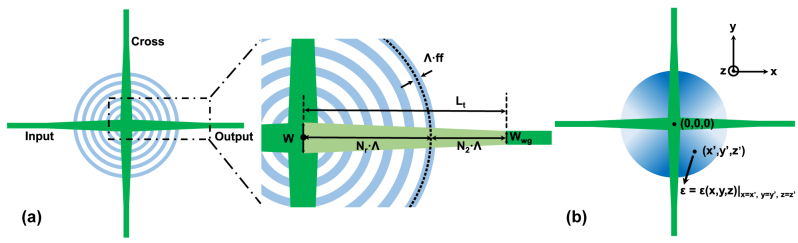
The schematics of the improved design. (a) The overall structure and specific parameter definitions. (b) The illustration of how the SWG is regarded as an anisotropic homogeneous medium with gradually changing relative permittivity tensors.

Hence, an improved design of SWG-based crossing is proposed. Instead of 1D SWG, we adopt a curved SWG to fill the lateral cladding region. As demonstrated in Figure 3(a), it basically consists of four tapered strip waveguides with length *L*_t_ for the four arms. The taper width alters linearly from *W*_wg_ to *W*, the same as the initial design. Then *N*_r_ rings with the same width of Λ ⋅ *ff*, all centered at the intersection (0,0), are placed. The center radius of the *n*th ring is defined as *r*_n_ = *n* ⋅Λ, so that the gaps between adjacent rings are equal. Note that a similar definition of *L*_t_ is applied compared with the initial design. The length is divided into two parts, with *N*_r_ and *N*_2_ grating period, respectively, although the second part does not contain any SWG structures. Therefore, it is defined that *L*_t_ = (*N*_r_ + *N*_2_)*Λ as illustrated in Figure 3(a).

Above is how the proposed curved SWG cladding is formed. Such structure mitigates both issues in the initial design. Firstly, it avoids the use of long tapers since the structure itself has a gradually-changing geometry. Meanwhile, the curved SWG can be treated as a medium with gradually varying permittivity. If we establish a Cartesian coordinate plane as in Figure 3(b), the relative permittivity tensor at point (*x*,*y*,*z*) can be written as Equation (8), following the conversion rule of second-order tensors under coordinate transformations:

(8) $$\begin{array}{cc} \varepsilon \left(x,y,z\right)= & \left[\begin{array}{ccc} \varepsilon^{\mathrm{xx}}\left(x,y\right) & \varepsilon^{\mathrm{xy}}\left(x,y\right) & 0\\ \varepsilon^{\mathrm{yx}}\left(x,y\right) & \varepsilon^{\mathrm{yy}}\left(x,y\right) & 0\\ 0 & 0 & \varepsilon^{\mathrm{zz}}\left(x,y\right) \end{array}\right],\\ z\in & \left(0,220\;nm\right), \end{array}$$

where:

(9) $$\left\{\begin{array}{cc} & \varepsilon^{\mathrm{xx}}\left(x,y\right)=n_{⊥}^{2}\cdot \cos^{2}\theta \left(x,y\right)+n_{\|}^{2}\cdot \sin^{2}\theta \left(x,y\right),\\ & \varepsilon^{\mathrm{yy}}\left(x,y\right)=n_{⊥}^{2}\cdot \sin^{2}\theta \left(x,y\right)+n_{\|}^{2}\cdot \cos^{2}\theta \left(x,y\right),\\ & \varepsilon^{\mathrm{xy}}\left(x,y\right)=\left(n_{⊥}^{2}-n_{\|}^{2}\right)\cdot \cos\theta \left(x,y\right)\cdot \sin\theta \left(x,y\right),\\ & \varepsilon^{\mathrm{yx}}\left(x,y\right)=\varepsilon^{\mathrm{xy}}\left(x,y\right),\\ & \varepsilon^{\mathrm{zz}}\left(x,y\right)=n_{\|}^{2}, \end{array}\right.$$

with *θ*(*x*, *y*) = arctan(*y*/*x*). Compared with the initial design, such a model avoids rapid transition of waveguide cross section while maintaining the same functionality.

PSO with large meshes is utilized again to get suboptimal parameters as the starting point of the follow-up parameters sweep. The target parameters and their bounds are listed in the Table 2. Note that the SWG period Λ is also considered in the PSO for an extra degree of freedom. The generation size is adjusted from 10 to 15 to avoid local optimums, while the number of generations is reduced from 30 to 25 to accelerate the optimization process.

**Table 2:** Ranges of parameters in PSO for the improved design.

Parameters	*N*_r_	*N*_2_	Λ (µm)	*ff*	*W* (µm)
Lower bounds	7	5	0.180	1/3	0.400
Upper bounds	25	20	0.220	2/3	0.900

**Figure 4:**
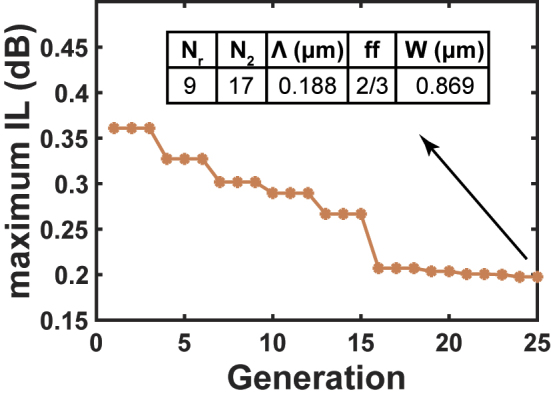
FOM trend of the PSO with the improved design.

The FOM trend during the PSO, i.e. the evolution of the maximum IL from the first to the last generation, is shown in Figure 4. After 25 generations, the maximum IL of the improved design over all the optical communication bands reaches 0.198 dB. With the improved design and extended parameter space, the optimized PSO demonstrates excellent optimization efficiency. The algorithm manages to decrease the FOM by 0.164 dB. The best parameter set after the convergence is listed in Figure 4 as well, which will be referred to in the following optimizations.

Same as the initial design, the *ff* also reaches the upper bound 2/3 after PSO. In the following step of optimization, although the SWG period Λ is no longer set as the constant value of 0.180 µm, the *ff* is still fixed as 2/3 for simplification. As for *N*_2_, it is mostly related to the length of the taper strip waveguide. Though it is possible to further improve the performance by extending the taper, this parameter is relatively independent of the other geometries. Therefore *N*_2_ is fixed at the suboptimal value of 17, so that compact size and good performance can be achieved simultaneously.

Consequently, deciding the best set of *N*_r_, *W*, and Λ becomes the next step. For the improved design, a more meticulous optimization flow is developed from here. Initially, a 3-D parameter sweep is conducted. The values are selected near the suboptimal parameters from the PSO listed in Figure 4. Specifically, *N*_r_ ∈ {7, 8, 9, 10, 11, 12, 13}, *W* ∈ {0.750, 0.800, 0.850, 0.900, 0.950} µm, and Λ ∈ {0.180, 0.185, 0.190} µm are chosen. 3-D FDTD simulations with denser meshes are performed, and the FOM remains the maximum IL.

**Figure 5:**
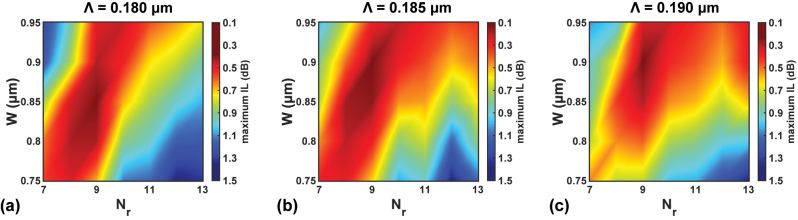
Calculated maximum IL as functions of *N*_r_ and *W* with (a) Λ = 0.180 µm, (b) Λ = 0.185 µm, and (c) Λ = 0.190 µm.

**Figure 6:**
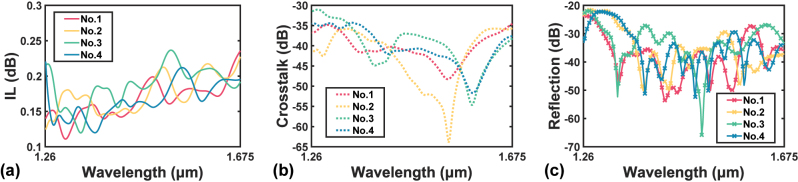
Calculated spectra of (a) IL, (b) crosstalk, and (c) reflection of all candidate geometries specified in Table 3.

The results are displayed in Figure 5. It is reasonable to choose the geometry with the least FOM and then finalize the design. However, since the mesh accuracy can still be increased and the crosstalk has not been considered yet, an extra step of optimization is introduced. A FOM threshold FOM_th_ is defined to select the best *k* sets of parameters, where *k* depends on FOM_th_. Then the structure is finalized by choosing the geometries with the best secondary FOM among the *k* candidate sets. In this work, for instance, FOM_th_ is set to 0.25 dB. Since only 4 sets of parameters fulfill FOM < 0.25 dB, they are selected as candidates and sent to the 3-D FDTD simulation engine again, but with finer meshes. The 4 sets of parameters are shown in Table 3, and their corresponding calculated spectra of IL, crosstalk, and reflection are demonstrated in Figure 6. One sees that all the candidates perform well in terms of the IL spectrum. Then the maximum crosstalk is chosen as the secondary FOM, and design no. 2 achieves the least maximum crosstalk. Hence, the geometry is determined that *N*_r_ = 9, *W* = 0.850 µm, and Λ = 0.185 µm. Under the highest mesh accuracy, the structure obtains max(IL) = 0.229 dB, max(Crosstalk) = −35.6 dB, and max(Reflection) = −21.6 dB, outperforming the initial design in terms of broadband performance except for the reflection property. One should notice that the footprint of the improved design is only 9.78 × 9.78 µm^2^, smaller than that of the initial design, 16.56 × 16.56 µm^2^. Furthermore, besides the centering SWG that takes 3.38 × 3.38 µm^2^ space, the rest of the area is rather empty since it only contains taper strip waveguides, which makes the component arrangement around the crossing very flexible when designing the circuit. Therefore, this is a very compact design.

**Table 3:** Candidate sets of parameters selected from Figure 5.

Parameters	*N*_r_	Λ (µm)	*W* (µm)
No. 1	9	0.180	0.850
No. 2	9	0.185	0.850
No. 3	9	0.185	0.900
No. 4	9	0.190	0.900

Table 4 concludes the three-step optimization for the improved design. The three steps are compared in terms of optimization method, simulation technique, mesh accuracy, simulation time, FOM, and number of iterations. Such a layered process avoids a large number of time-consuming simulations with dense meshes at one time, while maintaining the reliability of the optimization.

**Table 4:** Summary of the three-step optimization process.

Step	Method	Technique	Mesh accuracy	Simulation time	FOM	Number of iterations
1	PSO	3-D FDTD	∼10 mesh points/λ	Short	max(IL)	25 × 15
2	3-D sweeping	3-D FDTD	∼14 mesh points/λ	Medium	max(IL)	3 × 5 × 7
3	1-D sweeping	3-D FDTD	∼18 mesh points/λ	Long	max(Crosstalk)	4

**Figure 7:**
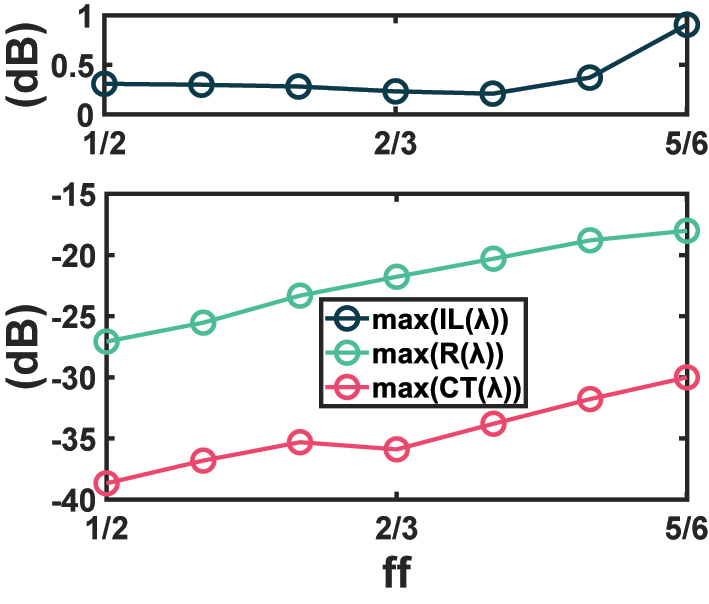
Dependence of maximum IL, maximum crosstalk, and maximum reflection on fill factor, as a fabrication tolerance analysis.

Last but not least, the fabrication tolerance of the improved design is discussed. Due to the periodic nature of SWG, the fill factor *ff* tends to be more sensitive to fabrication errors than the period Λ. Hence, our analysis focuses on the fill factor. The maximum IL, crosstalk, and reflection as functions of *ff* are calculated and plotted in Figure 7. It is implied from Figure 7 that the performance is not very tolerant to a largely increased *ff* since the maximum IL reaches around 1 dB when the fill factor increases from 2/3 to 5/6. Nevertheless, the design performs stably as *ff* declines, and the maximum crosstalk and maximum reflection even decrease. With a *ff* ranging from 1/2 to 7/9, the maximum IL over the 415-nm optical band is kept below 0.38 dB, the maximum CT is lower than −31 dB, and the maximum R is below −18 dB. It is then a reasonable choice to apply a slightly lower fill factor when producing the crossing on the chip, to acquire more tolerance of fabrication errors, lower crosstalk, and less reflection at the cost of marginally higher IL overall.

**Figure 8:**
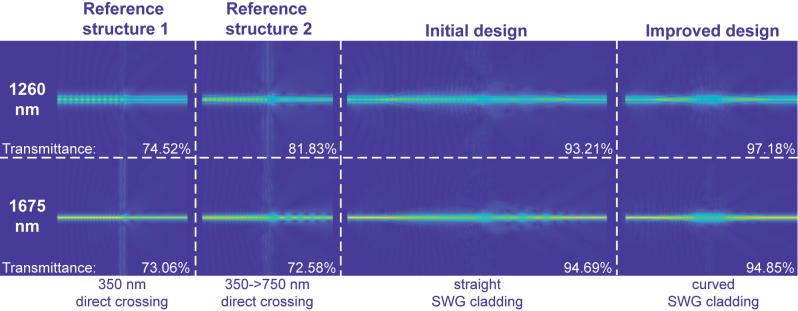
Electric field distribution, specifically $\left|E_{y}\right|$ of the two reference structures (Figure 2(c)), the initial design (Figure 1), and the improved design (Figure 3), at 1260 nm and 1675 nm.

To conclude the design process, we demonstrate the electric field distribution of the initial and the improved design, along with the two reference structures in Figure 2(c). The absolute values of the *y*-axis components of the electric field, $\left|E_{y}\right|$ at the *z* = 0 plane are captured from simulations and displayed in Figure 8. It is shown that the transmittance can be increased by the SWG cladding, especially the curved SWG, at both the lower and upper bounds of the wavelength range, namely 1260 nm and 1675 nm.

## 3 Experiment

**Figure 9:**
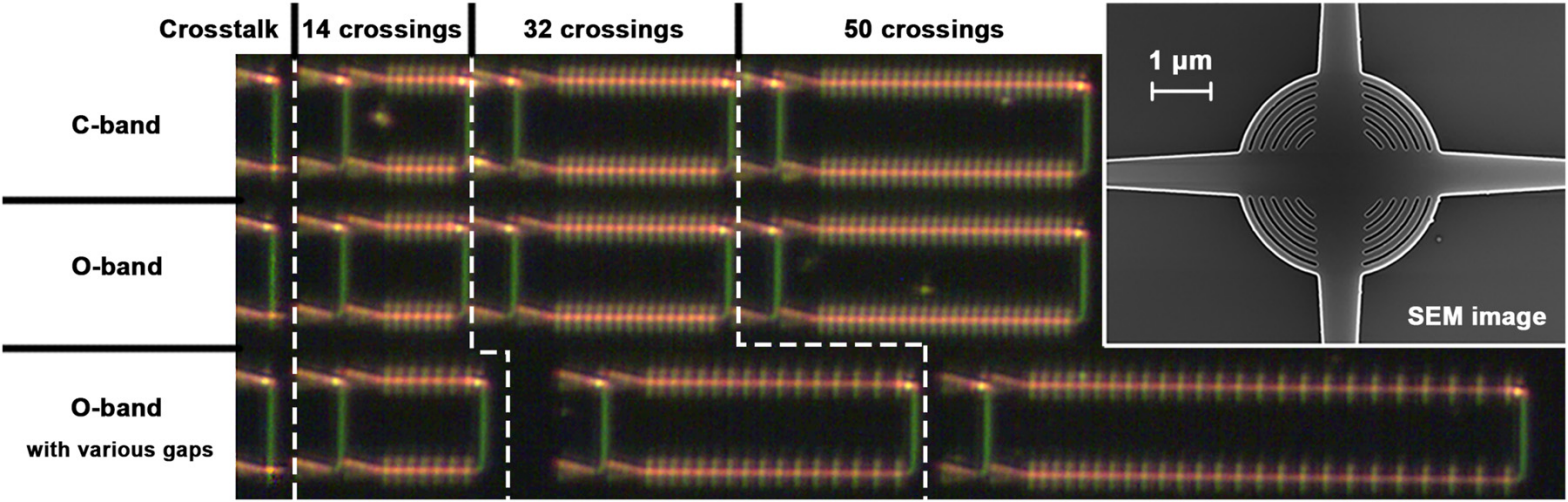
Measurement arrangement for the proposed crossing. Inset shows the SEM image of a single fabricated crossing with the improved design.

Both the initial and improved designs were fabricated on the standard 220 nm SOI platform with a 2.2 µm-thick top cladding oxide, by the NanoSOI fabrication process of Applied Nanotools Inc. Broadband vertical grating couplers are applied to couple light into and out of the chip [42, 43]. Since the calculated loss of the proposed device is too low to neglect the measurement errors, we measured the transmission spectrum of 14, 32, and 50 cascaded crossings to accurately estimate the insertion loss, as shown in Figure 9. The test structure is duplicated, but with O-band and C-band grating couplers respectively, so that the performance over [1260 nm, 1360 nm], and [1500 nm, 1630 nm] can both be measured. As for the crosstalk test, the cross port rather than the output port of the crossing is connected to the output grating coupler. Back-to-back connected pairs of grating couplers are placed near the devices for calibration.

The image of a single fabricated crossing with the improved design from the scanning electron microscope (SEM) is displayed as an inset of Figure 9. The clear features show that the curved SWG is decently fabricated.

**Figure 10:**
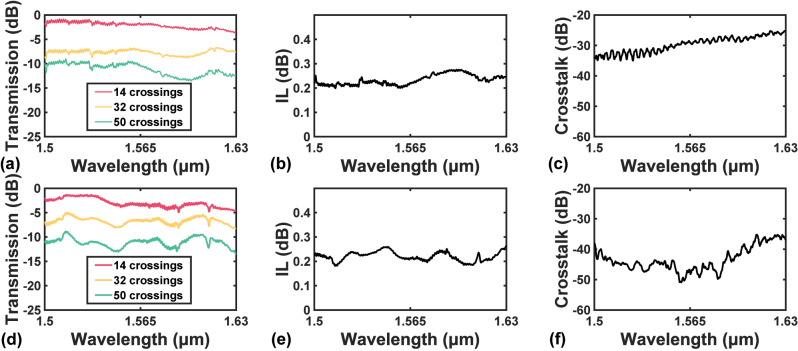
Experimental results near the C-band. (a) Measured transmission spectra with 14, 32, and 50 cascaded crossings, (b) fitted IL spectrum, and (c) measured crosstalk spectrum of the initial design from 1500 nm to 1630 nm. (d) Measured transmission spectra with 14, 32, and 50 cascaded crossings, (e) fitted IL spectrum, and (f) measured crosstalk spectrum of the improved design from 1500 nm to 1630 nm.

The experimental results from 1500 nm to 1630 nm are shown in Figure 10. For the initial design, the measured transmission spectra with various numbers of cascaded devices are shown in Figure 10(a), from which the ILs are extracted by linear fitting in Figure 10(b). To make sure that all three measured values are considered at each wavelength, the point (0,0) is also added, so that the fitted IL is based on four data points. Figure 10(c) displays the measured crosstalks over the measured wavelength range. Whilst Figure 10(d)–(f) corresponds to the results of the improved design in the same order as stated above. As can be seen from Figure 10(b) and (e), the IL response over [1500 nm, 1630 nm] ranges from 0.199 dB to 0.275 dB for the initial design, and from 0.181 dB to 0.264 dB for the improved design. Whilst the responses are both flat, the maximum measured IL of the improved design is 0.011 dB below that of the initial design. The improved design also outperforms the initial design on the crosstalk, as the maximum crosstalk of the improved design is only −35.2 dB, which is 10.0 dB below the maximum value for the initial design.

**Figure 11:**
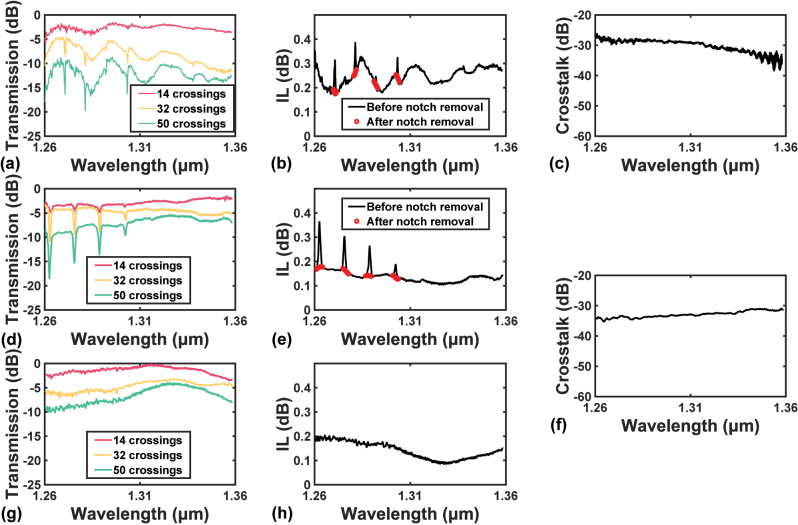
Experimental results in the O-band. (a) Measured transmission spectra with 14, 32, and 50 cascaded crossings, (b) fitted IL spectrum, and (c) measured crosstalk spectrum of the initial design from 1260 nm to 1360 nm. (d) Measured transmission spectra with 14, 32, and 50 cascaded crossings with uniform gaps, (e) fitted IL spectrum, and (f) measured crosstalk spectrum of the improved design from 1260 nm to 1360 nm. (g) Measured transmission spectra with 14, 32, and 50 cascaded crossings with various gaps and (h) fitted IL spectrum of the improved design from 1260 nm to 1360 nm.

The results obtained in the O-band are more complicated to analyze than that near C-band. From Figure 11(a) and (d), notches in transmission spectra are observed at wavelengths shorter than 1310 nm. Since the notches emerge uniformly on the wavelength axis, it is possible that they come from the response of Fabry-Pérot cavities. Tracing back to Figure 6(c), relatively high reflection is observed at the same wavelength range. The calculated reflections are larger than −23.7 dB for wavelengths shorter than 1310 nm, while they drop rapidly below −25.1 dB in wavelengths >1325 nm. Thus, multiple Fabry-Pérot cavities are formed between the cascaded crossings at short wavelengths. Although a single Fabry-Pérot cavity is not harmful due to the weak reflection, with uniform gaps between the fabricated devices, the responses of the multiple Fabry-Pérot cavities overlap and become spikier. This also explains why the notches in the spectrum with 50 crossings are deeper than that with 14 crossings.

Aware of the fact that the notches are not caused by the crossing itself, the ILs at the notches can be estimated by spline interpolation using the data points beside the notches, as demonstrated by the red dots in Figure 11(b) and (e). The maximum IL of the improved design is only 0.178 dB, much lower than that of the initial design, 0.352 dB. Comparing Figure 11(c) and (f), it is inferred that the improved design is also better than the initial design in terms of crosstalk in the O-band. The maximum measured crosstalk is −30.9 dB for the improved design and −26.2 dB for the initial design.

Although such estimation methodology is acceptable for the crossing to be used as a single component in a circuit, it does not make sense when multiple crossings are cascaded on the chip, just as the way the crossings are fabricated in our test chip. We propose two methods to mitigate this issue. The first method is to decrease the reflection. The condition for the gratings to operate in the SWG regime is given by *λ* > 2*n*_eff_Λ. Therefore, if the period of the SWG is further decreased, the devices will operate further away from the Bragg reflection regime. However, the minimum feature size also shrinks under such modification, adding challenges to the fabrication process. The *n*_eff_ can be decreased as well by reducing the fill factor *ff*. The effect has been predicted by Figure 7, where the maximum reflection declines when *ff* is smaller. Nonetheless, it comes at a cost of larger IL overall.

Hence, the second method is proposed. Instead of reducing the reflection, we change the way of arranging the cascaded crossings. As shown in Figure 9, we introduce various gaps between the cascaded crossing to prevent the responses from overlapping. The transmission spectra of such an arrangement are measured and displayed in Figure 11(g). The results prove that such a design manages to eliminate the notches in the spectra. As can be seen from Figure 11(h), the maximum IL is 0.204 dB, and the ILs at wavelengths <1310 nm are generally higher than that obtained from the uniformly placed crossings shown in Figure 11(e). Since the responses of the multiple Fabry-Pérot cavities are no longer overlapping, the increase in ILs can be regarded as those responses spread out on the spectrum. Because the results with various gaps are closer to the real situation and need less post-processing, they are considered as the final estimated IL spectrum of the improved design.

**Figure 12:**
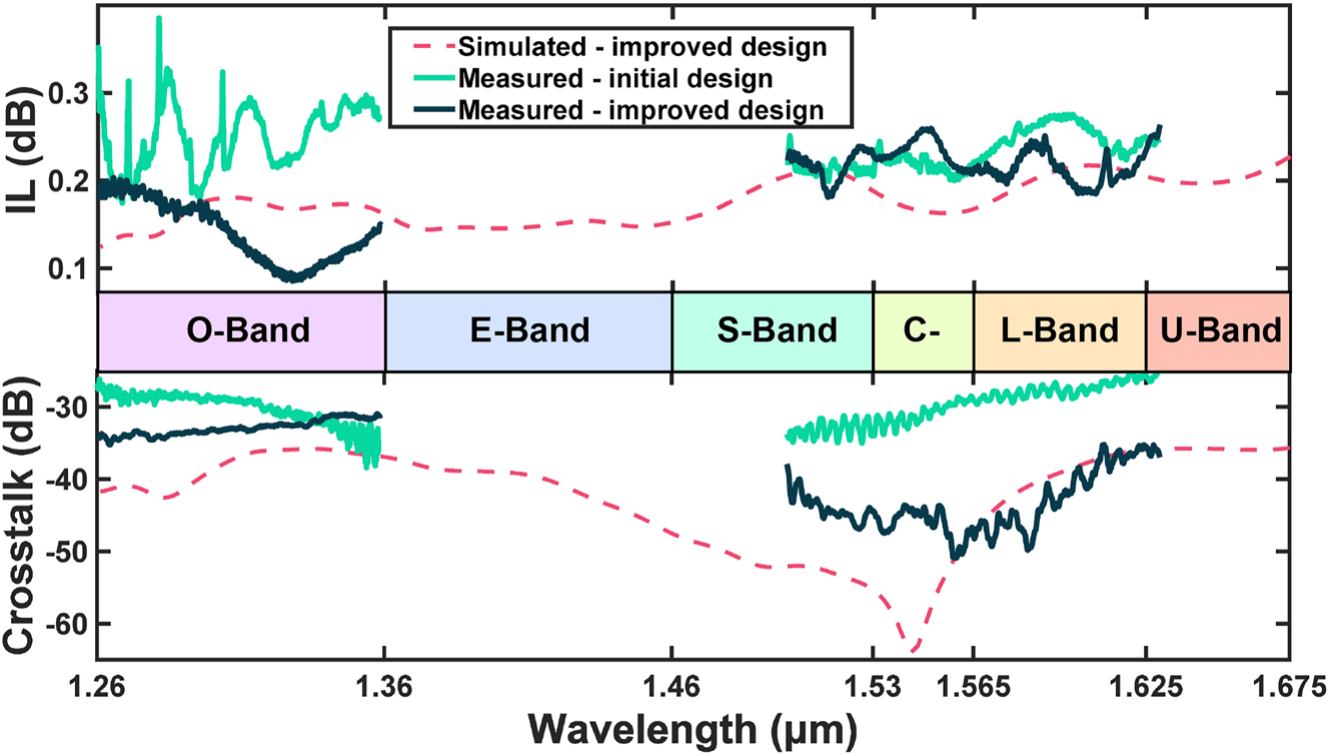
Multi-band device performance comparing the measured results of the improved design with the measured results of the initial design and the calculated results of the improved design, in terms of IL and crosstalk.

Finally, we conclude the overall performance of our proposed design in Figure 12. We compare the measured results of the improved and the initial design and the simulated results of the improved design. It can be implied that the measured IL and crosstalk spectra accord well with the simulation for the improved design, with slightly higher maximum IL and crosstalk. We also prove the superiority of the improved design compared to the initial design from an experimental perspective. Over the 230-nm wavelength range of measurement, the proposed device achieves a maximum IL of 0.264 dB and a maximum crosstalk of −30.9 dB, demonstrating decent functionality over essential optical communication bands including the O-, C-, and L-bands.

## 4 Analysis on temperature sensitivity

Aside from the broadband feature of our proposed designs that are demonstrated in the previous sections, the sensitivity of temperature should also be discussed. Typical low-loss crossing designs on SOI usually utilize the self-imaging effect of multi-mode interference to concentrate the light at the intersection. While this is an effective method, the beat length is sensitive to temperature changes, which leads to performance degradation in an environment with heat fluctuation. Very few works on waveguide crossing report temperature-related results.

On the contrary, our proposed design introduces the SWG as lateral claddings, and single-mode transmission is guaranteed through the crossing. Such simplicity may stabilize the device performance within a large range of temperatures. To evaluate it, the spectra of IL, crosstalk, and reflection are calculated using the index profile of silicon under 250 K, 293 K, and 350 K. The refractive index information is collected from [44]. For the oxide layers, since the thermal-optics coefficient of SiO_2_ is much lower than that of silicon, the index is kept the same. Note that the indices under 293 K are the profile applied in all previous simulations.

**Figure 13:**
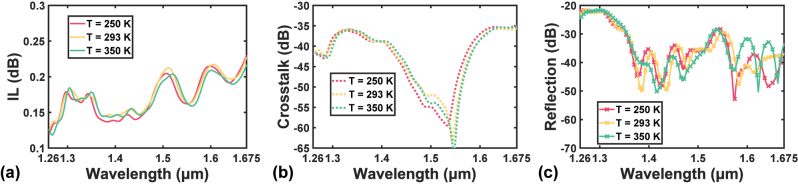
Calculated spectra of (a) IL, (b) crosstalk, and (c) reflection under various temperatures.

The calculated results shown in Figure 13 regarding the improved design exhibit no significant difference among the spectra under different temperatures. In Figure 13(a), the IL ranges from 0.124 dB to 0.229 dB over all optical communication bands under 293 K. Whilst the IL ranges are [0.120 dB, 0.232 dB] and [0.118 dB, 0.219 dB] for 250 K and 350 K, respectively. A closer inspection of the curves shows that the shape of the IL spectrum is maintained from 250 K to 350 K, only with a slight red shift as the temperature increases. A similar statement can be made on crosstalk and reflection. The maximum crosstalks are −34.9 dB, −35.6 dB, and 35.3 dB under 250 K, 293 K, and 350 K, respectively. As for reflection, the maximum values are −21.6 dB, −21.6 dB, and −21.7 dB for the three temperatures. The pattern of the spectrum is kept during temperature changes in both Figure 13(b) and (c).

**Figure 14:**
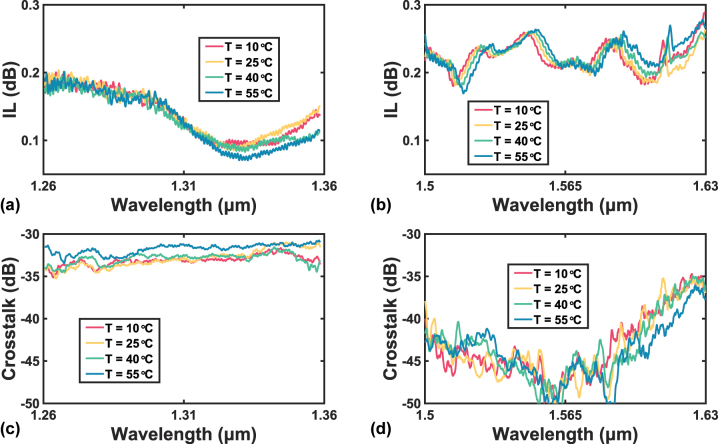
Measured IL spectra (a) from 1260 nm to 1360 nm, and (b) from 1500 nm to 1630 nm, measured crosstalk spectra (c) from 1260 nm to 1360 nm, and (d) from 1500 nm to 1630 nm of the improved design with various gaps under different temperatures.

Although the temperature insensitivity has been proved by simulations, it needs further verification from experiments. Since a thermoelectric cooler (TEC) is inserted under the testbed, a TEC controller can be used to tune the temperature of the chip. The transmission spectra are then repeatedly measured under different temperatures, including 10 °C, 25 °C, 40 °C, and 55 °C. The results are shown in Figure 14. From 10 °C to 55 °C, the ILs are kept below 0.204 dB from 1260 nm to 1360 nm, and they are below 0.289 dB from 1500 nm to 1630 nm. As for the crosstalk, the values are all below −30.7 dB from 1260 nm to 1360 nm, and they are below −34.7 dB from 1500 nm to 1630 nm. These values are very close to the results reported in the previous section. It is then inferred that the temperature hardly affects the device performance, which is an advantage for the crossing to be used in real-world circuits.

## 5 Conclusions

In conclusion, we have designed two versions of multi-band SOI waveguide crossing that apply SWG as lateral cladding. The initial design uses straight SWG cladding to reduce the index contrast, and then decrease the proportion of the light that is radiated out. However, the initial design cannot achieve a compact footprint due to the need of a long taper. Therefore, curved SWG is proposed to replace the straight waveguide, which forms the improved design. The curved SWG can be modeled as a metamaterial with gradually changing permittivity tensors, thus making the device exempt from the need for a long taper and improving the performance of the device as well. After a three-step optimization, the improved design achieves a calculated maximum IL of 0.229 dB and maximum crosstalk of −35.6 dB from 1260 nm to 1675 nm, covering all the optical communication bands. The proposed device is then fabricated and characterized over a 230-nm wavelength range consisting of [1260 nm, 1360 nm] and [1500 nm, 1630 nm]. To accurately estimate the IL, cascaded crossings with different numbers are fabricated on the chip, and the ILs are obtained by linear fitting. Since the relatively high reflection in the O-band causes notches in the IL spectrum, various gaps between the cascaded devices are introduced to cope with the issue. As a result, the improved design achieves measured maximum IL of 0.204 dB and maximum crosstalk of −30.9 dB from 1260 nm to 1360 nm, and maximum IL of 0.264 dB and maximum crosstalk of −35.2 dB from 1500 nm to 1630 nm. The temperature sensitivity of the proposed crossing has also been evaluated in simulation and experiments. Both calculated and measured results of the improved design under different temperatures demonstrate excellent performance stability. Such a broadband, compact, and temperature-insensitive design enriches the component library of multi-band PIC. Furthermore, the proposed curved SWG may provide a new perspective for designers to apply SWG in silicon photonics devices.
